# Evaluation of an integrated physical activity program for pregnant women: WELL-DONE! Study

**DOI:** 10.1093/eurpub/ckac131.449

**Published:** 2022-10-25

**Authors:** S Marini, A Masini, I Caravita, A Zannoner, F Scognamiglio, AA Coa, I Rescigno, G Sicari, G Castagna, L Dallolio

**Affiliations:** 1 QUVI, University of Bologna, Bologna, Italy; 2 DIBINEM, University of Bologna, Bologna, Italy; 3 DIMEC, University of Bologna, Bologna, Italy

## Abstract

**Background:**

Regular practice of physical activity (PA) during pregnancy has benefits for maternal and fetal health. Therefore, pregnant women (PW) should practice at least 150 minutes of moderate PA per week following the WHO guidelines. The aim of the study is to evaluate the effect of an adapted physical activity (APA) intervention for PW, to be included in childbirth preparation classes (CPCs) in terms of levels of PA, quality of life, physical performance, self-efficacy, sleep quality and anxious-depressive states.

**Methods:**

The WELL-DONE! Study is a quasi-experimental study conducted with pregnant women at St. Orsola hospital, Bologna. We compared an experimental group (EG) with a control group (CG). EG attended 1hour/week session of APA during the usual CPCs for a 6 weeks period, while the CG received a one hour lesson about PA recommendation in pregnancy. The pre-post evaluation was carried out through questionnaires and motor tests, to which PW were subjected at baseline (T0), after the intervention (T1) and 3 months after delivery (T2). We used the Pregnancy Physical Activity Questionnaire (PPAQ) to collect data regarding PA levels and sedentary behavior.

**Results:**

A sample of 50 pregnant women aged between 29-46 (mean age=35.44±3.99) was involved in the study (39 CG, 11 EG). After the intervention, PPAQ sedentary activity score was reduced in the EG group (-10.20±24.12) while remaining similar in the CG (0.58±22.65) without statistically significant differences between groups.

**Conclusions:**

Preliminary results of the study show a reduced sedentary time in PW, highlighting a positive trend in the EG. This data underlines that incorporating APA in the CPCs can be an effective and safe strategy. Nevertheless, further analysis must be needed to find out if this trend can be observed in light PA, in the moderate and vigorous one.

**Key messages:**

• Physical activity during pregnancy is a valuable tool for improving both mother and child well-being.

• Physical activity interventions, implemented in CPCs, seem useful in order to raise awareness about PA importance and reduce sedentarism in PW.

